# Supplementary material for: Electrochemical (Bio)Sensing Devices for Human-Microbiome-Related Biomarkers
Source: Sensors (Basel). 2023 Jan 11;23(2):837. doi: 10.3390/s23020837 (PMC9864681; doi:10.3390/s23020837)
Supplement: Supplementary file 1 [file sensors-23-00837-s001.zip › sensors-2119947-supplementary.pdf]

Table S1. Selected electrochemical (bio)sensors for biomarkers of human microbiomes and related biomolecules applied to clinical samples.

Microbiome	Biomarker	(Bio)sensor configuration	Technique	Analytical characteristics	Sample	Ref.
**Intestinal**	TMAO	PPy-MIP/ITO	DPV	DR: 1–15 μg mL^{-1}; LOD: 1 μg mL^{-1}	urine	23
	TMAO	enzyme TorA-FDH/MV/GCE	amperometry	DR: 2–110 μM; LOD: 2.96 nM	serum	24
	TMAO	enzyme TorA-GOD /Cat/MV/GCE	amperometry	DR: 2 μM–15 mM; LOD: 10 μM (serum)	10% serum	25
	TMAO	S. *loihica* PV-4/CCE	amperometry	DR: up to 250 μM; LOD: 5.96 μM	serum	26
	SCFAs	ZnO/PVA/AuE	EIS	DR: 0.5–20 mg mL^{-1}	bacterial isolates	33
	IL-10	microfluidic immunosensor: anti-IL-10 immobilized onto PCA/Gr-foam	EIS	DR: 10–100 fg mL^{-1}; LOD: 7.89 fg mL^{-1}	artificial saliva	48
	IL-1β<br>CRP	multiplexed wearable immunosensor: immobilization of anti-IL-1β and anti-CRP onto DTSSP-modified SPEs	EIS	DR: 0.2–200 pg mL^{-1} IL-1β<br>DR: 0.2 pg mL^{-1}–10 ng mL^{-1} CRP	spiked and on-body sweat	50
	CRP	Label-free immunosensor: anti-CRP-L-Cyst-AuNPs/SPE	amperometry	DR: 0.4–200 nM<br>LOD: 0.15 nM	serum	51
	CRP	Sandwich-type immunosensor: IrNPs/GO-DN-dAb-CRP-cAb-AuNPs/IL-MoS_2	amperometry	DR: 0.01–100 ng mL^{-1}<br>LOD: 3.3 pg mL^{-1}	serum	52
	MPO	Microfluidic device. cAb-biotin-Strep-MBs	amperometry	LOD: 0.004 ng mL^{-1}	plasma	57
	MPO	cAb-CuPdPt/GCE	amperometry	DR: 100 fg mL^{-1}–50 ng mL^{-1}; LOD: 33 fg mL^{-1}	serum	58
	MPO	Immunoassay: cAb adsorbed onto PS dipstick. Detection at N-CNTs/GCE	amperometry	DR: up to 700 μg mL^{-1}; LOD: 70 μg mL^{-1}	saliva	59
	ALP	Label-free immunosensor: anti-ALP/GO/Au-nano-dendroids/AuNPs/SPCE	EIS	DR: 100–1000 U L^{-1}; LOD: 9.1 U L^{-1}	serum	64
	ALP	DNA biosensor: AFC/ALP /MCH/ssDNA/AuE	DPV	DR: 20–100 mU mL^{-1}; LOD: 1.48 mU mL^{-1}	serum	65
	Indole	MWCNTs/CS/SPCE	DPV	DR: 5–100 μg L^{-1}; LOD: 0.5 μg L^{-1}	plasma	68
	5-HIAA	MIPPy/GCE	DPV	DR: 5×10^{-11}–5×10^{-5} M; LOD: 5×10^{-12} M	serum, urine, plasma	69
	iFABP	Label-free sandwich-type immunosensor: AuNPs-dAb-iFABP-cAb-interdigitated AuE	EIS	DR: up to 7 ng mL^{-1}; LOD: 0.68 ng mL^{-1}	urine	70
	CALP	Non-enzyme sandwich-like immunosensor: cAb-pDA /Au@MWCNTs/GCE. Detection with dAb-(PtNi@TCPP(Fe))	amperometry	DR: 200 fg mL^{-1}–50 ng mL^{-1}<br>LOD: 137.7 fg mL^{-1}	serum	72
**Oral**	MMP-9	Sandwich-type immunosensor: poly-HRP-bdAb-MMP-9-cAb-MBs/SPCE	amperometry (TMB)	DR: 0.03–2 ng mL^{-1}<br>LOD: 13 pg mL^{-1}	plasma	92
	MMP-9	Sandwich-type immunosensor: HRP-bdAb-MMP-9-cAb-MBs/SPCE	amperometry	DR: 8.0–75 pg mL^{-1}; 75–10000 pg mL^{-1}<br>LOD: 2.4 pg mL^{-1}	cell lysates<br>serum	93
	IL-8	Sandwich-type immunosensor: DI-3-b-Neu-bdAb-IL8-cAb-silane copolymer-ITO	Chronocoul. NADH/ $Os(bpy)_2Cl_2$	DR: 1 pg mL^{-1}–1 μg mL^{-1}<br>LOD: ~1 pg mL^{-1}	saliva	99

	IL-8	Label free immunosensor: IL-8-anti-IL-8-β-Ag_2MoO_4NPs/ITO	DPV	DR: 1 fg mL^{-1}–40 ng mL^{-1}<br>LOD: 90 pg mL^{-1}	saliva	100
	IL-8	anti-IL-8-AuNPs/rGO	DPV	DR: 500 fg mL^{-1}–4 ng mL^{-1}<br>LOD: 72.73 pg mL^{-1}	saliva	101
	IL-8	anti-IL-8-CB/PVDF/SPGMA/ITO	EIS	DR: 0.01–3 pg mL^{-1} ; LOD: 3.3 fg mL^{-1}	saliva, serum	102
	IL-8	Label-free immunosensor: anti-IL-8-PHA/ITO	EIS	DR: 0.02–3 pg mL^{-1}; LOD: 6 fg mL^{-1}	serum, saliva	103
	IL-8	Label-free immunosensor: anti-IL-8-IPTES/FTO	EIS	DR: 0.02–4 pg mL^{-1}; LOD: 11.9 fg mL^{-1}	serum, saliva	104
	IL-1β	Label-free immunosensor: anti-IL-1β-PHA/ITO	EIS	DR: 0.025–3 pg mL^{-1}; LOD: 7.5 fg mL^{-1}	serum, saliva	106
	IL-1β	Sandwich-type immunosensor: AP-strep-bdAb-IL-1β-cAb-IgG-ethynyl-azide-MWCNTs	DPV<br>(1-NPP)	DR: 10–200 pg mL^{-1} ; 200–1200 pg mL^{-1}<br>LOD: 5.2 pg mL^{-1}	saliva	107
	IL-8<br>IL-8 mRNA	HRP-strep-IL-8 mRNA-b-HCpIL-8-MBs/ and HRP-strep-bdAb-IL-8-cAb-MBs/SPdCE	amperometry	DR: 0.32–7.5 nM IL-8;<br>DR: 87.9–5,000 pg mL^{-1}IL-8 mRNA<br>LOD: 72.4 pg mL^{-1} IL-8; 0.21 nM IL-8 mRNA	saliva	108
	IL-1β<br>TNF-α	Dual immunosensor: poly-HRP-bcAb- IL1β- dAb- and poly-HRP-bcAb-TNFα -dAb-phe-DWCNTs/SPdCE	amperometry	DR:0.5–100 pg mL^{-1} IL-1β;1–200 pg mL^{-1} TNF-α,<br>LOD: 0.38 pg mL^{-1} IL-1β; 0.85 pg mL^{-1} TNF-α	serum, saliva	109
	anti-CCP	Label-free immunosensor: PANI/AuNPs/anti-CCP-CCP/ PANI/MoS_2/SPCE	SWV	DR: 0.25–1500 IU mL^{-1}<br>LOD: 0.16 IU mL^{-1}	10% serum	113
	RF<br>anti-CCP	Dual immunosensor: HRP- IgM-RF-Fc(IgG)-cMBs/ and HRP-IgG-anti.CCP-CCP-biotin-Neutr-MBs/SPdCE	amperometry	DR: 3–300 IU mL^{-1} RF; 10–1000 IU mL^{-1}anti-CCP<br>LOD: 0.8 IU mL^{-1} RF; 2.5 IU mL^{-1}anti-CCP	serum	113
**Nasal**	IL-8	Label-free immunosensor: cAb-CBMA/AuE	EIS	DR: 55 fM–55 nM; LOD: 1 fM	NELF	116
	IL-6	Non-enzyme sandwich-type immunosensor: NB-GO-dAb-IL-6-cAb-GO/AuE	SWV	DR: 1–300 pg mL^{-1}; LOD: 1 pg mL^{-1}	RAW cells;<br>live mice brain	123
	VEGF<br>IFN-γ<br>TNF-α	Multiplexed aptasensor: biotin-VEGF-apt-AQ-, biotin-IFN-γ-apt-MBT and biotin-TNF-α-apt-Fc-Strep/GO/AuE	SWV	DR: 5–300 pg mL^{-1}(VEGF, IFN-γ); 5–200 pg mL^{-1} (TNF-α); LOD: 5 pg mL^{-1}	serum, sweat	124
	VEGF	Ratiometric aptasensors: Fc-apt-biotin-strep -MB/GO-ph-GCE or -MB/GO/GCE	SWV<br>(I_{MB}/I_{Fc})	DR: 20–500 pg mL^{-1}; LOD: 7 pg mL^{-1}<br>DR: 10–500 pg mL^{-1}; LOD: 1 pfg mL^{-1}	serum	125
	EGFR<br>VEGF	Sandwich immunosensors: LP@Cd(II)-anti-EGFR- and LP@Cu(II)-anti-VEGF-MIP-DSP-SPAuE	PSA	DR: 0.05–50000 pg mL^{-1}; LOD: 0.01 pg mL^{-1}<br>DR: 0.01–7000 pg mL^{-1}; LOD: 0.005 pg mL^{-1}	serum	126

Keywords: AFC, aminoferrocene; ALP, alkaline phosphatase; Cat, catalase; CB, carbon black; CCE, carbon cloth electrode; CCP. cyclic citrulinated protein; CS, chitosan; DN, 1,5-diaminonaphthalene; DPV, differential pulse voltammetry; DR, dynamic range; DSP, 3,3′-dithiodipropionic acid di(N-hydroxysuccinimide ester; EGFR, epidermal growth factor receptor; EIS, electrochemical impedance spectroscopy; Fc, ferrocene; FDH, formate dehydrogenase; iFABP, intestinal fatty-acid binding protein; IFN-γ, interferon gamma; IL, 1-aminopropyl-3-methylimidazolium chloride; IPTES, 3-(triethoxysilyl) propyl isocyanate; ITO, indium-tin oxide electrode; LOD, detection limit; LP, liposome; MB: methylene blue; MCH, 6-mercapto-1-hexanol; MIP, molecularly imprinted polymer; MIPPy, molecularly imprinted polypyrrole; MV, methyl viologen; NB, Nile blue; N-CNTs, nitrogen-doped carbon nanotubes; pDA, poly(dopamine); PPy, poly(pyrrole); PS, polystyrene; PSA, potentiometric

stripping analysis; PVA, poly(vinylalcohol); PVDF, polyvinylidene fluoride; RF, rheumatoid factor; rGO, reduced graphene oxide; *S. Ioihica*, *Shewanella loihica*; SCFAs, short-chain fatty acids; SPGMA, shaped poly(glycicylmethacrylate); TCPP, tetrakis (4-carboxyphenyl) porphyrin; TMAO, trimethylamine N-oxide; TNF-α, tumor necrosis factor-α; TorA, trimethylamine N-oxide reductase; VEGF, vascular endothelial growth factor.
